# Supplementary material for: Genome-wide identification and expression analysis of calmodulin-like proteins in cucumber
Source: PeerJ. 2023 Jan 13;11:e14637. doi: 10.7717/peerj.14637 (PMC9841910; doi:10.7717/peerj.14637)
Supplement: Supplemental Information 1 [file peerj-11-14637-s001.docx]

Table S1 Primers used for qRT-PCR

| Gene name | Forward primer | Reverse primer |
| --- | --- | --- |
| *CsCML1* | AGTTCCTCCGTGAACCTTCC | ACGCCTTCAGAATTCCCCTG |
| *CsCML2* | AGAGGTTTTGCAGAAGGGGG | TCACATAGCCATCGCCATCAT |
| *CsCML3* | GGCAGGGTGTGTGCAAGATA | AGCTCGTCGCAGGAAATGAA |
| *CsCML4* | TGGGGTGTCTGGAGGAAGAT | GCCAACACGTTTTCGAGTCC |
| *CsCML5* | ACGGCGATGGCTACATTGAT | TGCAATTCCTCGGCAGTGAT |
| *CsCML6* | TTCACACACGCCAATCCAGT | ACTTTGCCGTCGGAATCTGT |
| *CsCML7* | GCAGTCTCACCCAACTCGAA | GAATTCGATGGAGCCGTTGC |
| *CsCML8* | ATGATCAGCGGCGTCGATAG | TTTCGTCGGAGACAGAACGG |
| *CsCML9* | TCGTGATGCTCGTGGTTCAA | TCTGCTCTCGACTCAAAGGT |
| *CsCML10* | GGATCGCGTCAGAGGAAGAG | CTCAAACGTCTCCCGCATCT |
| *CsCML11* | ATGATGGAGGAAGGTGACGC | TCCCCATCAATCGAAGCCAC |
| *CsCML12* | CTTTCTCCCGACCTACCGAG | TCGAAATCCTTCTCGCCGAT |
| *CsCML13* | GAGCTCGCCAGTCACTCAAA | ACAGCGTAATCAAGAAGGCGA |
| *CsCML14* | ACCGGAATGATCAGGGAAGC | TTGCCATCCAGGTAACACCC |
| *CsCML15* | CGAAACCGGAGATGGGGAAA | CTCCTCGGCGATTATGGCTT |
| *CsCML16* | TTCACCCGTTGGGTTGGAAT | GGCCAACAGAGTGCAGATGA |
| *CsCML17* | TCTGGCACACATTGATTTTCTCT | AGCTTCGCATCCAGTCTGTT |
| *CsCML18* | GATCAACCTCGGGGAGAAGC | TCATCCAACGGCCATCATCA |
| *CsCML19* | GTGAACTCAGCGGCAACATC | AGGTCGATGAAACCGTCACC |
| *CsCML20* | ATGGCAAGATCTCGGCAACA | CGTCCATCATCCTCTTGGCA |
| *CsCML21* | GGGAGTGTATAGCGGTGGTG | CCGTCCACGAATCTCACGAA |
| *CsCML22* | ACATATCCGCCACCGAACTC | GCGAGCGAATCAACAACTCC |
| *CsCML23* | GATGGTTTGCTGTGTGTGGG | TGAACCCGCAACCTTCACTA |
| *CsCML24* | GGAGTGTGGTTGAAGAATTGGC | GTCACCGTTCGTGTTTGTCG |
| *CsCML25* | GCCTCTTGGCTCTGTTCCTC | TCTTCCGCTGACCCAAACAG |
| *CsCML26* | CGACAAGAACGACGATGGGT | CCCCTTTCAACATTTCCTCCG |
| *CsCML27* | GGGAGTTCTTCAATCTCATGGC | TCAGCTTCATTGACCATTTGCT |
| *CsCML28* | GCTGAGCCATGTCTACTGGA | CAACGTGGCCATCACCATTC |
| *CsCML29* | CAAGCGCATAGCAAAGGAGC | CACGATCTCGATCAGCCTCA |
| *CsCML30* | GCTTGGCTGGAAGCAAAGAG | AATCCACGAATCCATCGCCA |
| *CsCML31* | GCGCCTTCAAGTATTTGGGC | AAGCTGATGACATCGTCGCA |
| *CsCML32* | ACAGCTCGCCTGGATTTTCA | CCATCGTGGTTTGCATCGAC |
| *CsCML33* | GCCTTCGGTATGATGGGTTCA | CGCCATCTTCATCAGCACAAG |
| *CsCML34* | TGGAACCCTCACCAAGCAAG | AGACTCTCTTGCGGGTTTGAA |
| *CsCML35* | TGCTTCTCACCATCACCAACA | CCTCATCTTGTACGGCCCAC |
| *CsCML36* | GGCTCTTCCTGGTGTAAGCA | AGGCCGTCACCATTCTCATC |
| *CsCML37* | CGACCCCACTCTCTCTCGTA | GCACCCATCGTCTCCCATAG |
| *CsCML38* | TTGGGATGAGGGAAAACGGG | CCGCCAACTCGAACTTTTCC |
| *CsCML39* | GATGGGAAACTCTCTCCGCC | AACCCATCACCGTCCGAATC |
| *CsCML40* | GATGGTTTGCTGTGTGTGGG | TGAACCCGCAACCTTCACTA |
| *CsCML41* | TAAACCTTCTACGTCGGGGC | CTCCAATCACACTCCGAGCA |
| *CsCML42* | ACGAAGAACTCCGCAAGGTT | AGGAAGTGAGAACGGCATCG |
| *CsCML43* | TGCTGCACAAAAATCGTGGG | TCCTCAAACATCCCCTCAAGC |
| *CsCML44* | AATCCCAAACGTCATGGGGG | CATACGCGGTTGCTTGCTTC |
| Actin | TTCTGGTGATGGTGTGAGTC | GGCAGTGGTGGTGAACATG |
